# Biomechanical optimization of the magnesium alloy bionic cannulated screw for stabilizing femoral neck fractures: a finite element analysis

**DOI:** 10.3389/fbioe.2024.1448527

**Published:** 2024-08-30

**Authors:** Yunwei Cui, Kai Ding, Hongzhi Lv, Xiaodong Cheng, Zixi Fan, Dacheng Sun, Yifan Zhang, Wei Chen, Yingze Zhang

**Affiliations:** ^1^ Department of orthopaedic surgery, Hebei Orthopaedic Clinical Research Center, Hebei Medical University Third Hospital, Shijiazhuang, China; ^2^ Key Laboratory of Biomechanics of Hebei Province, Orthopaedic Research Institute of Hebei Province, Shijiazhuang, China; ^3^ NHC Key Laboratory of Intelligent Orthopaedic Equipment, Hebei Medical University Third Hospital, Shijiazhuang, China; ^4^ Engineering Research Center of Orthopaedic Minimally Invasive Intelligent Equipment, Ministry of Education, Shijiazhuang, China; ^5^ Chinese Academy of Engineering, Bingjiaokou Hutong, Bejing, China

**Keywords:** magnesium alloy bionic cannulated screw, finite element analysis, bionic hole, optimal diameter, implantation direction

## Abstract

**Purposes:**

The magnesium alloy bionic cannulated screw (MABCS) was designed in a previous study promoting cortical–cancellous biphasic healing of femoral neck fractures. The main purpose was to analyze the bore diameters that satisfy the torsion standards and further analyze the optimal pore and implantation direction for stabilizing femoral neck fractures.

**Methods:**

The MABCS design with bionic holes with a screw diameter of less than 20% met the torsion standard for metal screws. The MABCS was utilized to repair the femoral neck fracture via Abaqus 6.14 software, which simulated the various stages of fracture healing to identify the optimal biomechanical environment for bionic hole size (5%, 10%, 15%, and 20%) and implantation direction (0°, 45°, 90°, and 135°).

**Results:**

The stress distribution of the MABCS fracture fixation model is significantly improved with an implantation orientation of 90°. The MABCS with a bionic hole and a screw diameter of 10% provides optimal stress distribution compared with the bionic cannulated screw with diameters of 5%, 15%, and 20%. In addition, the cannulated screw fixation model with a 10% bionic hole size has optimal bone stress distribution and better internal fixation than the MABCS fixation models with 5%, 15%, and 20% screw diameters.

**Conclusion:**

In summary, the MABCS with 10% screw diameter bionic holes has favorable biomechanical characteristics for stabilizing femoral neck fractures. This study provides a biomechanical foundation for further optimization of the bionic cannulated screw.

## Introduction

With more and more traffic accidents and an aging population, 1.7 million femoral neck fractures are estimated to occur per year worldwide, which is approximately 3.4% of all fractures (Florschutz et al., 2015; Major and North, 2016; Chen et al., 2017; Ding et al., 2024). Given the low cost and minimal invasiveness of the procedure, closed reduction and multiple cannulated screws (CSs) are the recommended treatment for young-to-middle femoral neck fractures (Florschutz et al., 2015; Li and Cole, 2015). Nevertheless, postoperative complications of CSs, including osteonecrosis of the femoral head (ONFH) and nonunion, have been reported to reach 50%, which can impact life quality and increase the economic burden (Li and Cole, 2015). Therefore, new treatment concepts and methods have been proposed to address the treatment dilemma of femoral neck fractures, and the novel configuration and materials of the cannulated screw structure have been improved.

The anatomical structure of the proximal femur and the mechanism of fracture healing are closely related to the occurrence of postoperative ONFH and nonunion, which is predominantly due to the complexity of reconstructing the proximal femoral trabecular system (Ding et al., 2021b). The placement process of internal fixation often damages part of the trabecular bone. In addition, the traditional internal fixation also occupies a considerable intraosseous volume, which hinders the reconstruction of the trabecular bone. Thus, some scholars suggested that additional bionic holes in cannulated screws provide physical space for trabecular bone regeneration around internal fixation, which has improved the biomechanical properties and promotes fracture healing (Wang et al., 2023). Furthermore, abnormal trabecular reconstruction related to the absorption of the surrounding trabeculae occurred due to stress masking caused by a large difference in the elastic modulus between conventional implants and trabeculae (Li et al., 2021). To address these issues, our team proposed the concept of bionic fixation of fractures and designed a magnesium alloy bionic cannulated screw (MABCS), drawing upon biomechanical principles, imaging data, and clinical research. This approach facilitates trabecular bone growth through bionic holes, restoring bone continuity and promoting a biphasic healing process in intertrochanteric fractures (Wang et al., 2020). The low elastic modulus of the magnesium alloy significantly diminishes the likelihood of bone stress concentration and the incidence of complications such as ONFH (Zhao et al., 2016; Tian et al., 2018; Cun et al., 2020; Ding et al., 2021b; Li et al., 2021). However, there is little evidence of the optimal biomechanical environment for the bionic hole size and implantation direction.

Given the above concept, finite element analysis was used to determine bionic cannulated screws that identify the optimal configuration of bionic holes for stabilizing femoral neck fractures, providing references for the clinical application of MABCSs.

## Materials and methods

This study was approved by the Ethics Committee of the Third Hospital of Hebei Medical University. Written informed consent was obtained from the volunteering subject.

**Screening diameters and implantation orientations of the bionic cannulated screw for optimal biomechanical characterization of fixed femoral neck fractures**

**FIGURE 1:**
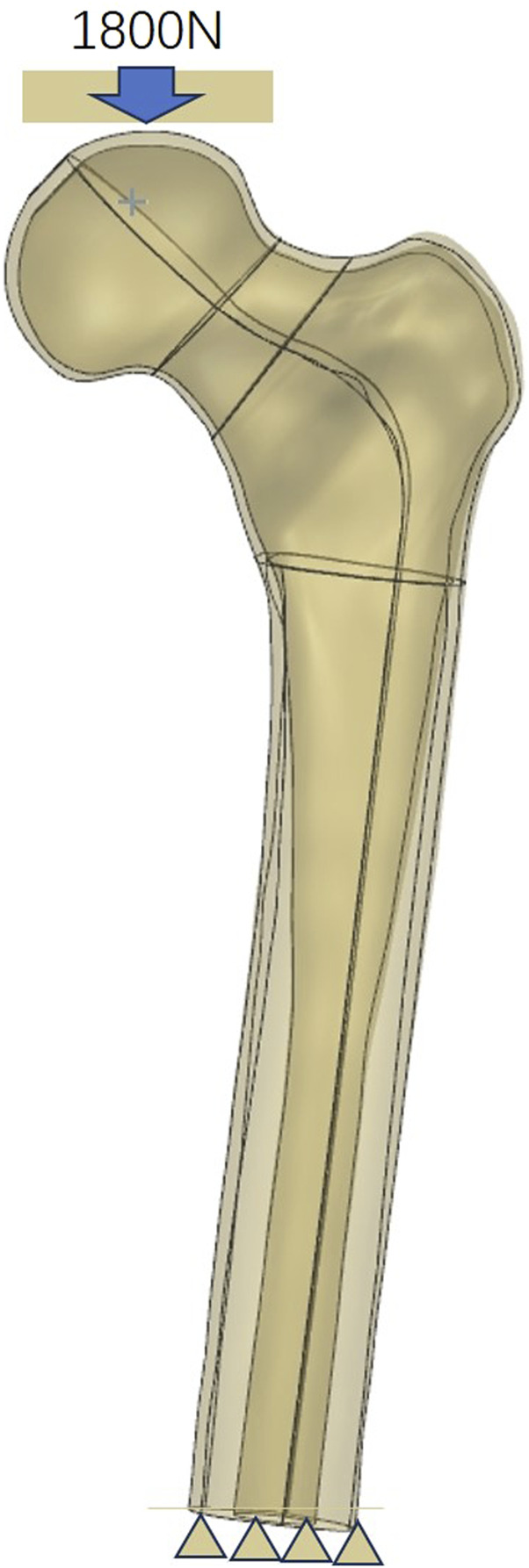
Boundary conditions of the fracture fixation models.

**FIGURE 2:**
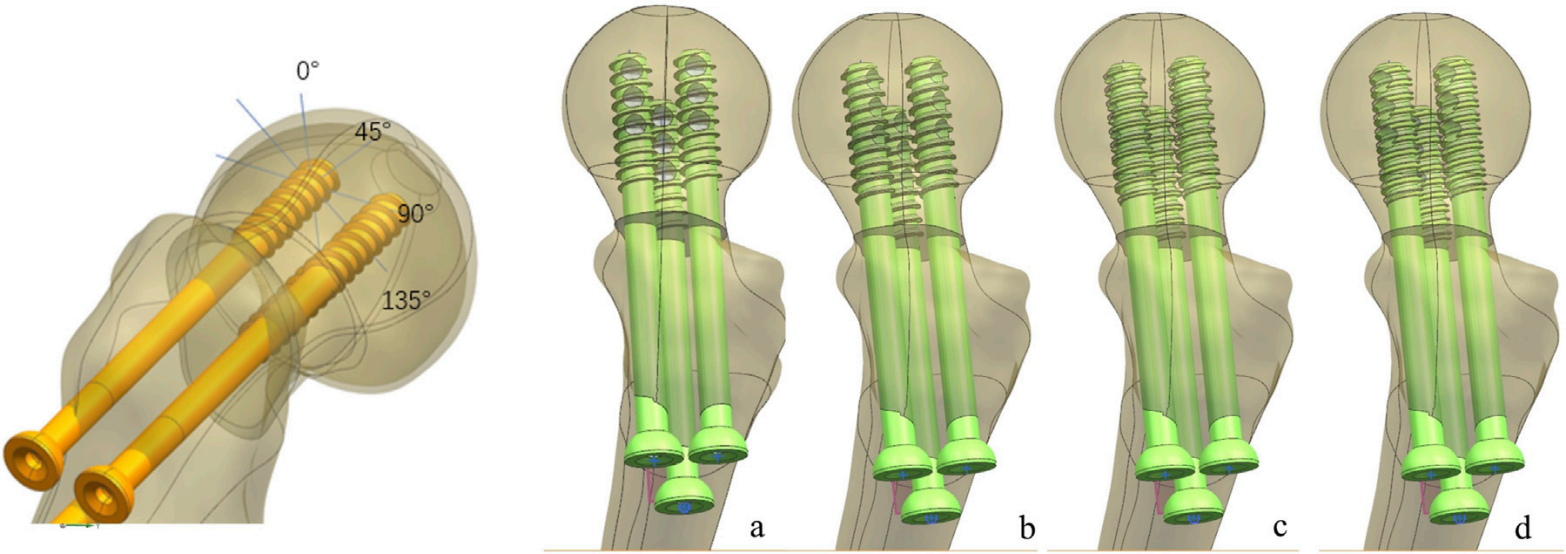
Fracture fixation models of the MABCS with different implantation directions. **(A)**, 0°; **(B)**, 45°; **(C)**, 90°; and **(D)**, 135°.

**FIGURE 3:**
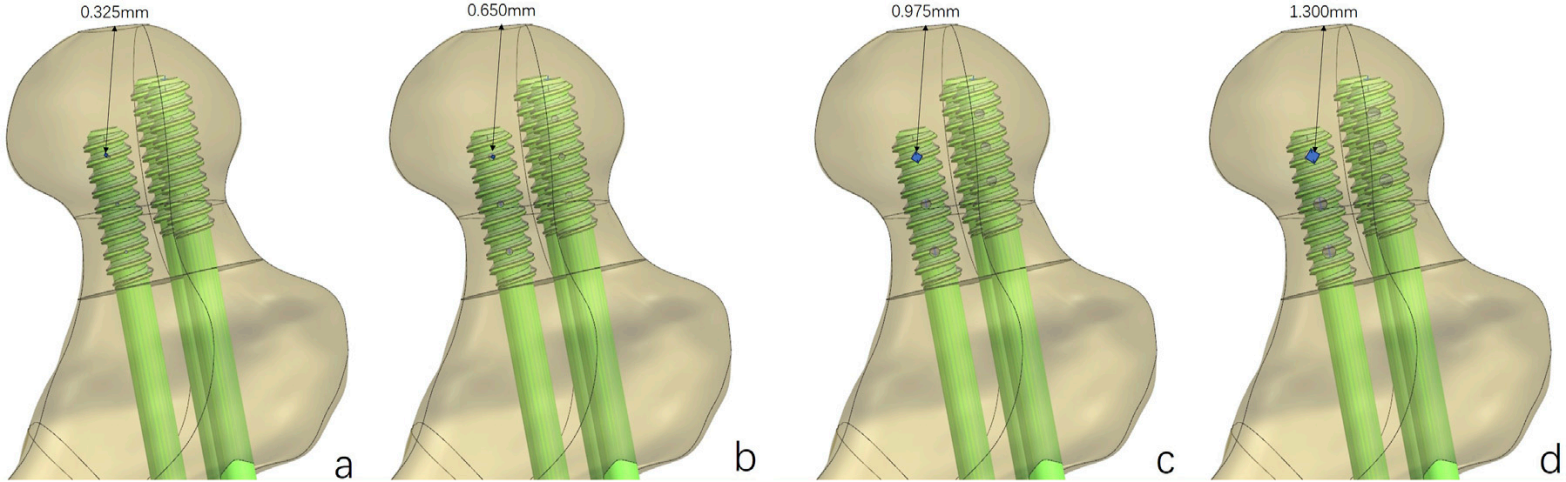
Fracture fixation models of the MABCS with different pore sizes. Bionic holes with 5% **(A)**, 10% **(B)**, 15% **(C)**, and 20% **(D)** screw diameters.

One healthy male volunteer (height: 170 cm; body weight: 60 kg) was included in this study. The lower extremities were used to scan for constructing proximal femur models using Mimics 21.0, and then Geomagic 2013 was used to construct the solid proximal femur model. An unstable femoral neck fracture (Pauwel’s angle of 50°) was constructed and fixed with a MABCS with bionic holes of a 25% diameter at different directional angles, including 0°, 45°, 90°, and 135°, using UG software (Figures 1–3). The reference plane was taken as the line from the femoral head to the distal femoral stem and the screw centerline, and the centerline of the bionic hole was set to 0° to the reference plane. The MABCS was rotated 45°, 90°, and 135° from the screw centerline to create different torsion models.

Based on the above experimental results, the implantation direction was set as 90°. Bionic holes with 5%, 10%, 15%, and 20% screw diameters were designed.

The above models were imported into HyperMesh 2013 to construct a C3D4 tetrahedral mesh, which was subsequently imported into Abaqus software. All the models were assigned linear elasticity and isotropy with the corresponding modulus of elasticity and Poisson’s ratio as reference (Ding et al., 2021b; Li et al., 2021) (Table 1). The relationship between the screw thread/bone and cortical screw/bone was set as tied. In addition, other bone–screw and bone–bone interfaces were assumed to have a surface-to-surface contact relation with a friction coefficient of 0.3 (Ding et al., 2021a). The joint load was set to 1,800 N (approximately three times the body weight) to simulate the single-leg stance of the human body, and the distal femur was set to 0 degrees of freedom in six directions (Li et al., 2019). The three-dimensional proximal femoral FEM, whose convergence analysis and effectiveness have been verified in previous studies, was used in this study (Ding et al., 2022; Ding et al., 2023; Zhang et al., 2023) (Figure 1).

**TABLE 1:** Material properties of all models in this study.

Model	Material	Young’s modulus (GPa)	Poisson’s ratio
Cortical bone	Cortical bone	17	0.3
Cancellous bone	Cancellous bone	1.5	0.3
MABCS	Mg alloy	45	0.316
MABCS (PO 3 months)	Mg alloy	36	0.316
MABCS (PO 12 months)	Mg alloy	9	0.316

**PO**, postoperative.

### Simulation of the fracture healing process

According to the experimental design, different contact relations were set up to simulate fracture healing periods. These periods were divided into three categories: fracture, part-healing, and fully healing. At the initial fracture stage, the contact relationship between the fracture surfaces was set to sliding and separation to simulate the compression reduction of the fracture. Furthermore, the 3-month and 12-month post-fracture models, respectively, were used to simulate bone ingrowth in this study. The cancellous bone contact relationship was set as tied without slip or displacement, in the part-healing period (postoperative 3 months). In addition, no slip or displacement of the cortical and cancellous bones were simulated (postoperative 12 months). The von Mises and displacement distributions of femoral neck fractures fixed with different-diameter cannulated screws at different stages of fracture healing were collected.

## Results

### Optimal implantation orientation of bionic cannulated screws for femoral neck fracture fixation

Under axial load, the peak von Mises stresses of the MABCS fixation model with a torsion angle of 90° were 278.1, 248.8, and 87.4 MPa for the three periods, respectively. These values represented 84.6%–98.7% of the other torsion angle MABCS fixation model for the fracture period, 98.4%–99.2% for the part-healing period, and 32.0%–87.6% for the fully-healing period.

**FIGURE 4:**
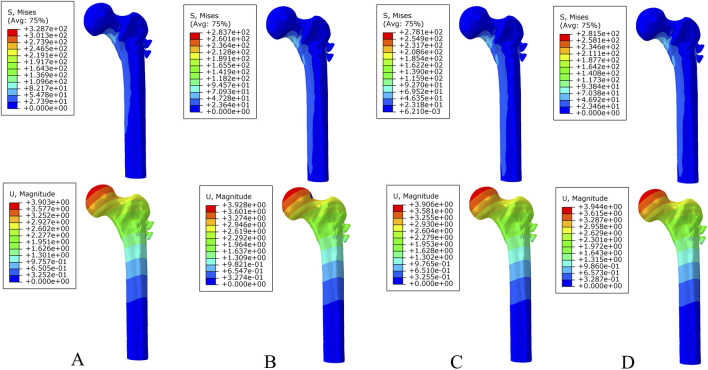
The von Mises stress and displacement distribution of 25%-bionic hole-diameter MABCS fixation models with different implantation directions at a non-healed fracture. **(A)**, 0°; **(B)**, 45°; **(C)**, 90°; and **(D)**, 135°.

**FIGURE 5:**
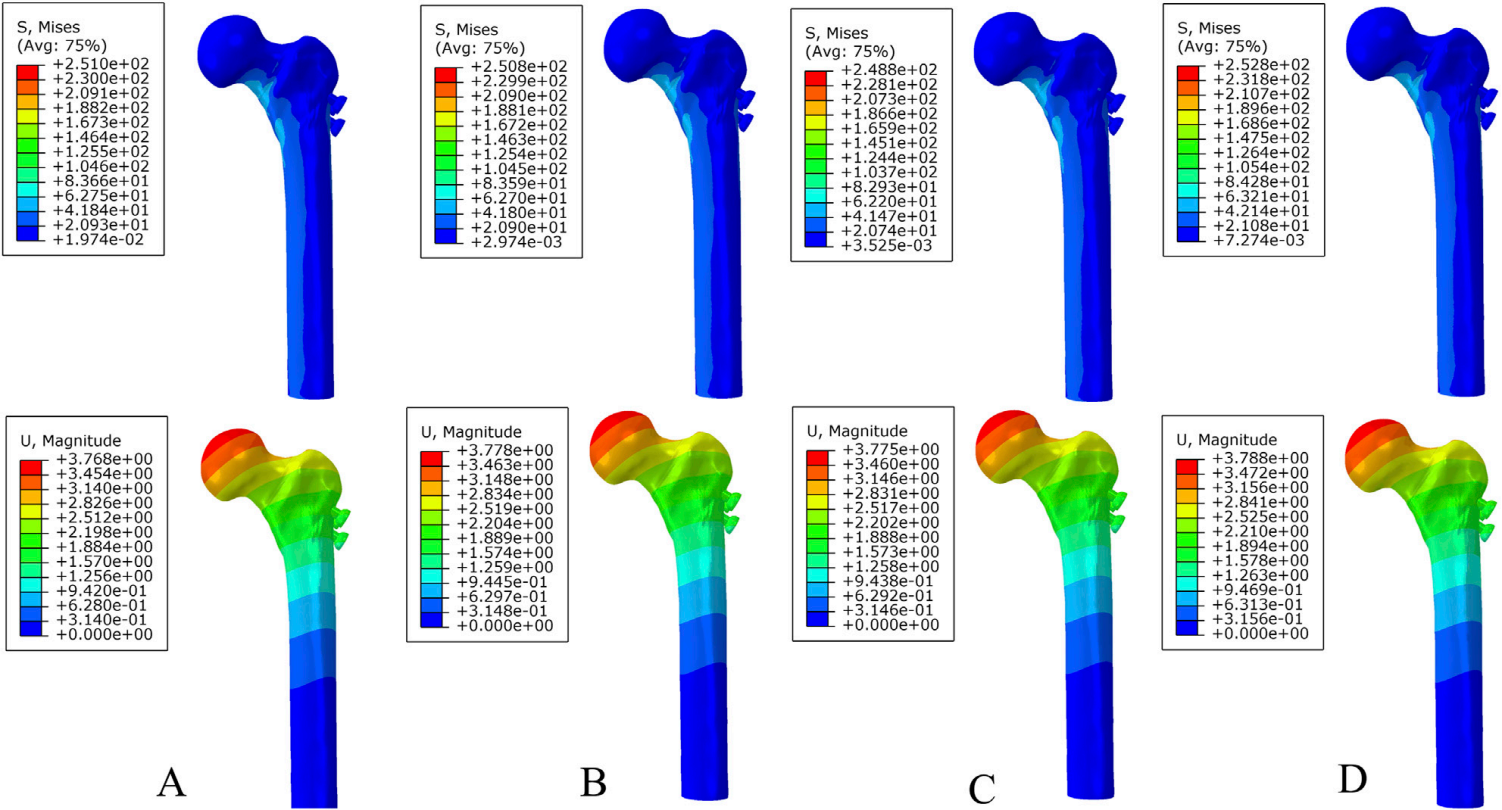
The von Mises stress and displacement distribution of 25%-bionic hole-diameter MABCS fixation models with different implantation directions at a partly healed fracture. **(A)**, 0°; **(B)**, 45°; **(C)**, 90°; and **(D)**, 135°.

**FIGURE 6:**
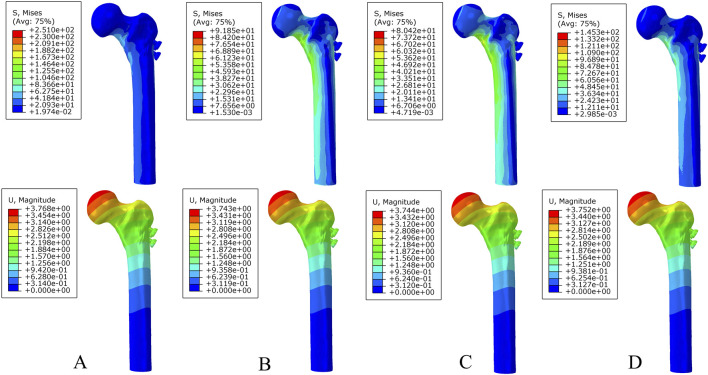
The von Mises stress and displacement distribution of 25%-bionic hole-diameter MABCS fixation models with different implantation directions at a fully healed fracture. **(A)**, 0°; **(B)**, 45°; **(C)**, 90°; and **(D)**, 135°.

**FIGURE 7:**
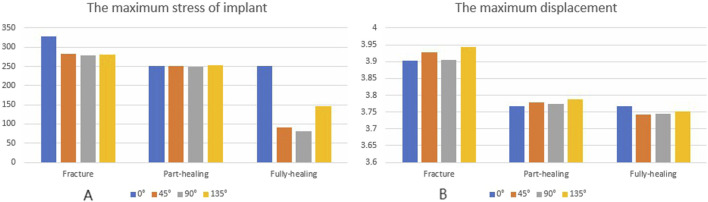
The von Mises stress **(A)** and displacement distribution **(B)** of 25%-bionic hole-diameter MABCS models with different implanted directions for three periods.

Additionally, the maximum displacement with a torsion angle of 90° was 3.9, 3.8, and 3.7 mm for the three periods, which is 99.0%–100.2% of the torsion angle fixation model. The implantation direction of bionic holes had no significant impact on the stability of fracture fixation during the healing process in the MABCS (Figures 4–7).

### Optimal bionic hole size for femoral neck fracture fixation

**FIGURE 8:**
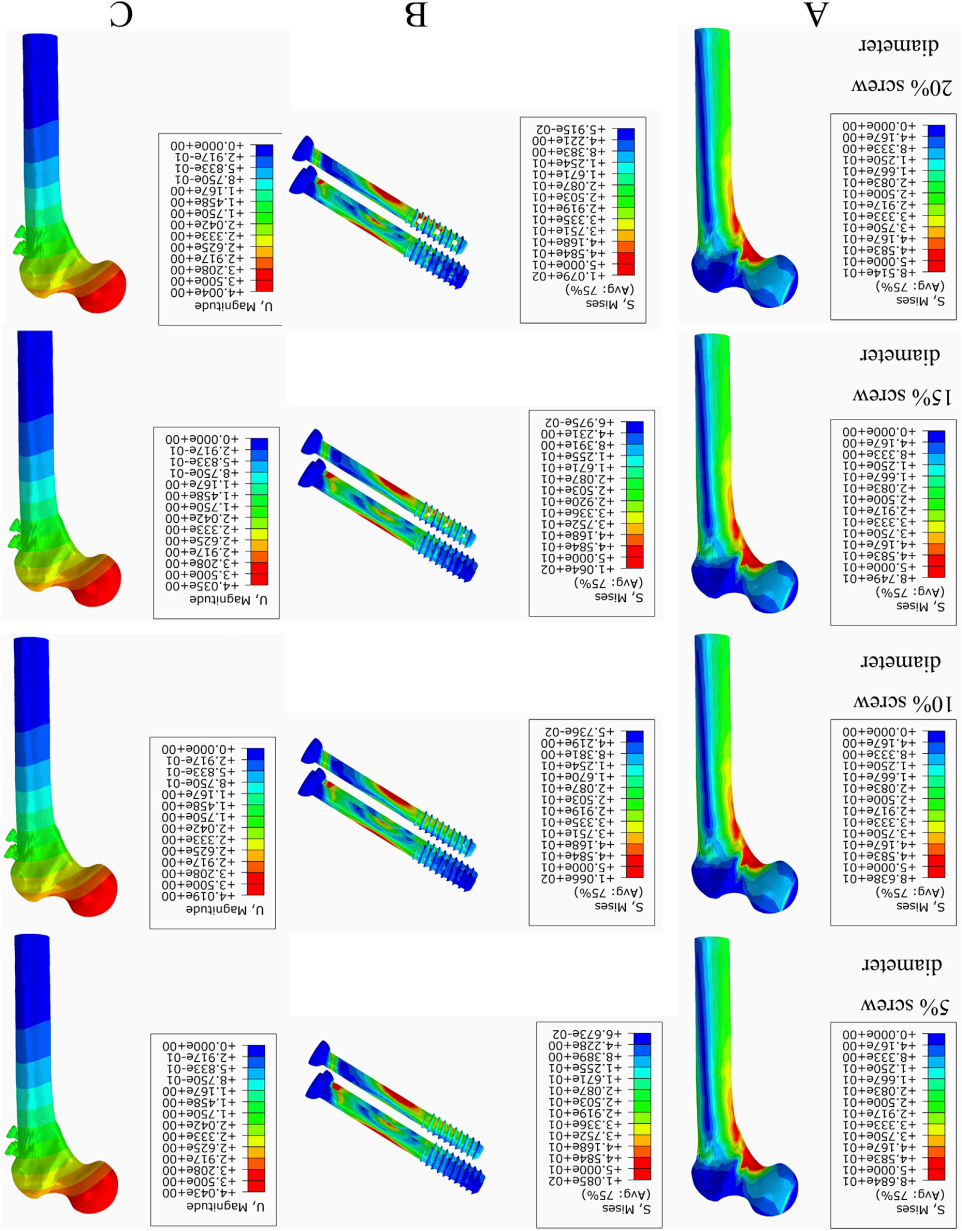
The von Mises stress and displacement distribution of MABCS fixation models with different pore sizes at a non-healed fracture with a torsion angle of 90°. Stress distribution of bone **(A)** and implants **(B).** Displacement of fracture models **(C)**.

**FIGURE 9:**
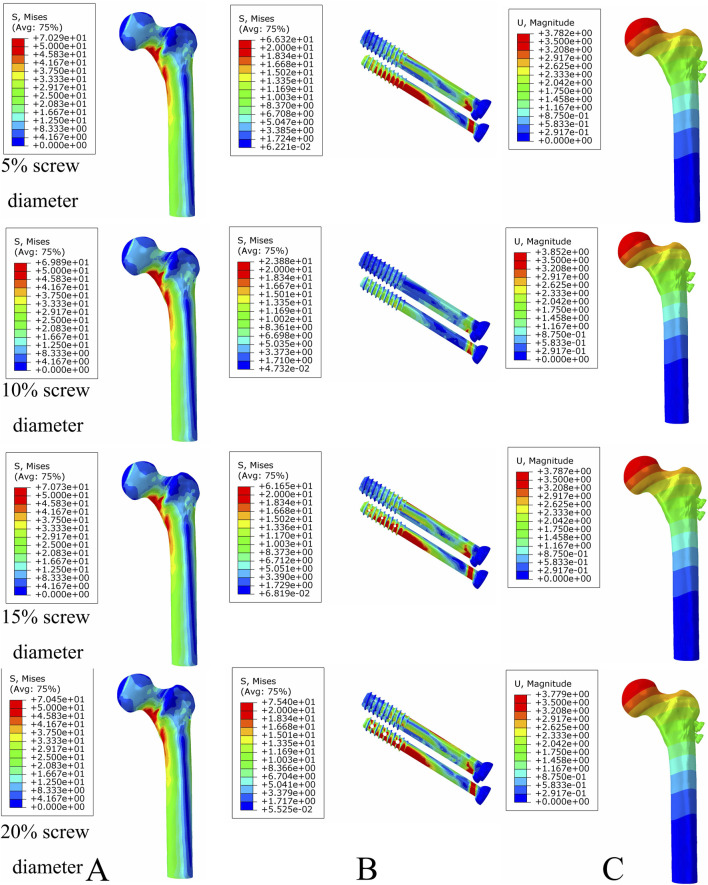
The von Mises stress and displacement distribution of MABCS fixation models with different pore sizes at a partly healed fracture with a torsion angle of 90°. Stress distribution of bone **(A)** and implants **(B).** Displacement of fracture models **(C)**.

**FIGURE 10:**
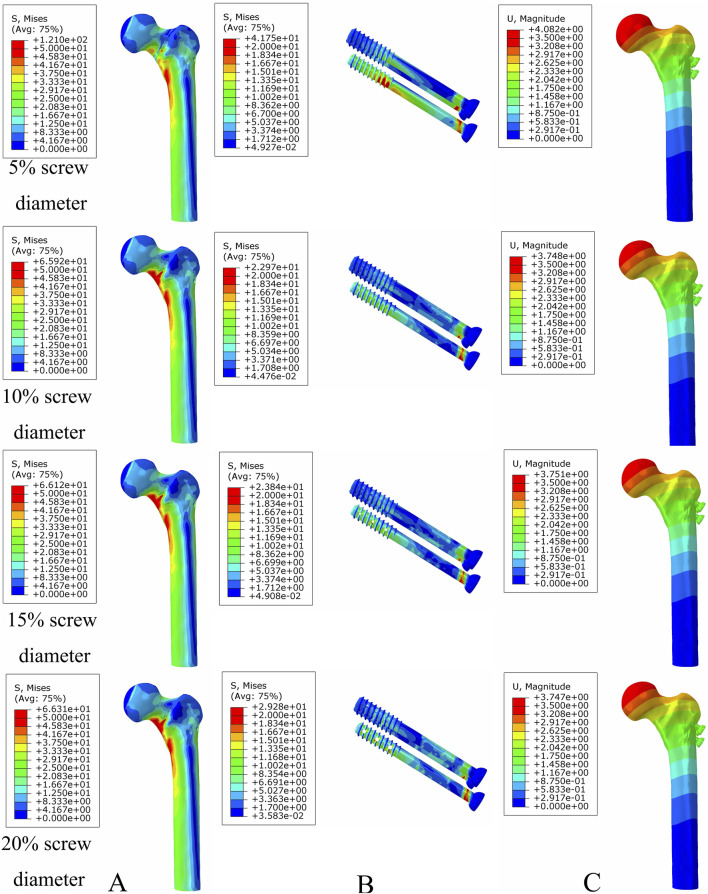
The von Mises stress and displacement distribution of MABCS fixation models with different pore sizes at a fully healed fracture with a torsion angle of 90°. Stress distribution of bone **(A)** and implants **(B).** Displacement of fracture models **(C)**.

**FIGURE 11:**
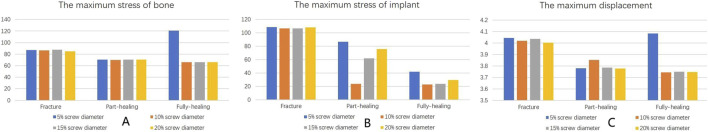
The von Mises stress of bone **(A)** and implants **(B)** and displacement distribution **(C)** of the MABCS with different pore sizes for three periods with a torsion angle of 90°.

Under axial load, the bone and internal fixation peak von Mises stresses of the MABCS with a bionic hole diameter of 10% at the early fracture, part-healing period, and fully healing period were 83.3 and 106.6 MPa, 69.6 and 23.9 MPa, and 65.9 and 23.0 MPa, respectively. These values represented 98.7%–101.5% and 98.2%–100.2% of other aperture size MABCS fixation models for the fracture period, 98.8%–99.4% and 27.6%–38.7% for the part-healing period, and 54.5%–99.7% and 55.0%–96.4% for the fully healing period. Furthermore, femoral neck fracture fixation stability gradually increased with the bionic hole enlargement (Figures 8–11).

## Discussion

This study revealed that the optimal stress distribution for MABCS fixation of femoral neck fractures is achieved when the bore size is 10% of the screw diameter at 90° to the direction of implantation. The results provide biomechanical guidance for potential clinical applications of bionic cannulated screws in the treatment of femoral neck fractures.

The MABCS has unique biomechanical properties. First, the increase in pore size has reduced the maximum displacement of the femoral neck fracture fixation models with a torsion angle of 90°, which results in the gradual enhancement of fracture stiffness. This may be related to the fact that an increase in the trabecular reconstruction volume enhances the internal fixation of holding forces, improving fracture fixation stability. Second, the biomechanical properties of the MABCS deteriorated with increasing bionic hole diameter. However, the biomechanical properties of bionic cannulated screws with a screw diameter of less than 20% met the criteria. The MABCS with a 10% bionic hole size (650 μm) for treating femoral neck fractures resulted in the best internal fixation and bone stress distribution, as verified by animal experiments (500–650 µm). The 10% bionic hole size (650 μm) provides an optimal balance between stability and stress distribution, as well as a favorable biomechanical environment for trabecular reconstruction (Collins et al.). The study posits that the addition of a bionic hole may facilitate the reconstruction of trabecular bone; however, it may also compromise the mechanical properties of the screw, potentially increasing the risk of fixation failure (Li et al., 2021). It can be seen that there is an optimal interval between the maintenance of the trabecular bone reconstruction and the stress distribution in the MABCS fixation model. The results demonstrate that the main reason for the superior stress distribution of the bionic cannulated screw with a 10% screw diameter is due to its diameter being the most appropriate for this particular model, which is consistent with the results of animal experiments (Collins et al., 2021). Finally, the direction of screw implantation can also affect the biomechanical properties of the MABCS fracture fixation model, and the stress concentration is significantly improved in all cases with a torsion angle of 90°. The upper and lower portions of the bionic hole can bear the proximal femoral load and effectively transfer the load to the femoral lateral cortex. Our findings may be similar to those of the previous literature, suggesting that the requirements of stability and improved retention are conducive to bionic screws with a torsion angle of 90°(Qi et al., 2013).

In addition to the biomechanical properties, the bionic hole provides a physical space for fracture healing factors, thus improving the fracture fixation of the cannulated screw. Lv et al. designed a side-hole structure cannulated screw loaded with a recombinant human BMP-2-targeted slow-release system that promoted bone tissue regeneration (Houcheng et al., 2014). Hofmann-Fliri et al. attempted to design side holes on a cannulated screw and then injected bone cement into the cannulated screw through the end of the nail after implantation, which formed a contact surface with the bone at the bottom side holes, enhanced screw implantation stability and reduced the displacement and dislodgement of cannulated screws in the postoperative period (Hofmann-Fliri et al., 2016). Furthermore, the study identified the biomechanical references for this type of internal fixation, including embedding direction, bionic hole number distribution, pore diameter, and other key factors. However, the above treatments overcome the volume occupancy effects and still fail to reduce the existing postoperative complications due to the stress masking effect and the difficulty in removing the conventional porous materials. In response to this, the MABCS was designed for the treatment of femoral neck fractures with the aim of improving stress concentration, reducing complications, and providing physical space for the healing factors. The results of this study show that the stress on the bone and internal fixation was significantly reduced during the MABCS degradation , which provided a good biomechanical environment for ingrowth. This study did not directly describe the relationship between MABCS degradation and bone ingrowth, including degradation products, thread shape, pitch, and surface roughness, and further studies are needed.

Previous studies have demonstrated that the MABCS improves stress distribution and reduces stress-masking and volume-occupancy effects while maintaining stability (Xue et al., 2022). Based on the above studies, this study investigated the design of the MABCS in accordance with torsion standards and investigated the optimal bionic hole size and implantation direction to provide a biomechanical basis for the clinical application of the MABCS for femoral neck fractures. Compared to other medical metal materials, magnesium alloys are closer to human bone in terms of Young’s modulus and density, have better biocompatibility, and are less susceptible to degradation (Nabiyouni et al., 2018; Parai and Bandyopadhyay-Ghosh, 2019). In addition, the degradation of magnesium alloys releases magnesium ions, which can reduce the inflammatory response, promote osteogenic healing, and accelerate the process of fracture healing. At present, magnesium alloys have been used in rabbit ulna and mandible fracture models, respectively, indicating good fracture healing (Witte et al., 2007; Sun et al., 2016; Chen et al., 2022). In addition, magnesium alloys have been partially applied to the human body. Magnesium alloy nails are comparable to titanium nails in randomized controlled clinical trials for bunions (Windhagen et al., 2013). Furthermore, magnesium alloys have been used in femoral head necrosis, their clinical efficacy is reliable, and compared with traditional surgery, the procedure is simple and short, and the magnesium alloy screw can degrade by itself (Cheng et al., 2022). From the perspective of clinical practice, magnesium alloys have shown unique advantages in fracture treatment and bone defect repair as a potential biodegradable metal material (Chen et al., 2014). They have a similar density and elastic modulus to human bone, which helps reduce the stress-shielding effect and promote bone healing (Kamrani and Fleck, 2019). Clinical trials of magnesium alloy screws have been conducted, demonstrating favorable biocompatibility and mechanical properties, as well as uniform and predictable degradation characteristics (Saris et al., 2000; Lee et al., 2016). Using magnesium alloy bone nails can avoid the need for secondary surgery, reducing the economic and physical burden on patients (Hou et al., 2019). However, it is still necessary to consider the degradation rate to meet the needs of fracture healing in different populations and to ensure its safety and effectiveness.

Finite element analysis was used to simulate the degradation kinetics of screw materials and the fracture healing process to simulate the biomechanical properties of MABCS fixation of femoral neck fractures. According to the previous literature (Ding et al., 2021b; Li et al., 2021), we selected three time nodes in the fracture healing process as fracture separation period, trabecular bone healing period, and complete healing. The time nodes were 0 months, 3 months, and 12 months, respectively, and the corresponding elastic moduli of the degradable magnesium alloy were 45 MPa, 36 MPa, and 9 MPa, respectively (Cun et al., 2020). In addition, 3 months after fracture healing, we added trabecular bone ingrowth to simulate trabecular bone reconstruction. As a result, the finite element simulation, the fracture surface contact properties of different periods, the MABCS elastic modulus, and the trabecular bone ingrowth, in turn, changed the complete process of the simulated MABCS fixed femoral neck fracture.

This study has several shortcomings. First, the study used a finite element analysis to simulate the MABCS fixation of a femoral neck fracture, but there was no experiment with magnesium alloy, which may have had a potential influence on the reliability of the experiment. This study did not consider the above experimental changes, which may affect the results of this study. Second, the material properties of the MABCS used in this study were obtained from the previous literature, and the degradation rate of the MABCS was not tested, which may affect the reliability of the experimental results. Finally, the construction method of the fracture model in this study was planar, and the real differences in fracture morphology may affect the reliability of the fracture model (Zhan et al., 2024).

In conclusion, the MABCS with a 10% bionic pore diameter implanted in a 90° orientation can meet the stability of early fracture fixation with optimal stress distribution and provide a good biomechanical environment for trabecular bone reconstruction. The results of this study can provide a biomechanical reference basis for the clinical application of bionic magnesium alloys.

## Data Availability

The original contributions presented in the study are included in the article/Supplementary Material; further inquiries can be directed to the corresponding authors.
